# Dynamic frailty assessment predicts in-hospital and early post-discharge mortality in older patients hospitalized with acute heart failure: a prospective cohort study

**DOI:** 10.1186/s12877-026-07530-7

**Published:** 2026-04-20

**Authors:** Roberto Daniel Cortés Pestana, Helen Margarita Valenzuela Leal, Emma Puigoriol Juvanteny, Emilia Teresa Bernabeu Garri, Nuria Molist-Brunet

**Affiliations:** 1 Department of Geriatrics, Hospital Universitari Germans Trias i Pujol, Carretera de Canyet, s/n, Badalona, Barcelona, 08916 Spain; 2 University of Vic – Central University of Catalonia (UVIC-UCC), Vic, Spain; 3 Department of Cardiology, Hospital Universitari de Vic, Vic, Spain; 4 Laboratory of Tissue Repair and Regeneration (TR2Lab), University of Vic – Central University of Catalonia (UVIC-UCC), Fundació Hospital Universitari de La Santa Creu de Vic, and Hospital Universitari de Vic, Vic, 08500 Spain; 5 Department of Epidemiology and Statistics, Hospital Universitari de Vic, Vic, Spain; 6 Multidisciplinary Inflamation Research Group (MIRG), Institut de Recerca I Innovació en Ciéncies de la Vida i de la Salut a la Catalunya Central (IRIS-CC), Vic, Catalonia 08500 Spain; 7 Home Hospitalization Unit, Universitary Vinalopó Hospital, Elche, Spain; 8 RE-FiT BCN (Recerca en Envelliment, Fragilitat I Transicions a Barcelona), Parc Sanitari Pere Virgili, Barcelona, Spain; 9 Autonomous University of Barcelona, Barcelona, Spain

**Keywords:** Acute heart failure, Frailty, In-hospital mortality, Post-discharge mortality, Prognosis, Risk stratification

## Abstract

**Background:**

Frailty is insufficiently captured by conventional mortality risk scores in acute heart failure (AHF), particularly regarding its dynamic changes during hospitalization. We aimed to evaluate the relationship between different frailty assessment tools and in-hospital and 30-day post-discharge mortality in older patients hospitalized for AHF.

**Methods:**

We conducted a prospective cohort study (HOP-AHF cohort) in a second-level hospital between January 2023 and February 2024. Patients aged *≥* 80 years admitted for AHF to Acute Geriatric, Cardiology and Internal Medicine Wards were included. Frailty was assessed at baseline, within the first 48 h of admission, and at discharge using the Frail Index–VIG (FI-VIG), FRAIL scale, Clinical Frailty Scale (CFS), Tilburg Frailty Indicator (TFI) and Identification of Seniors at Risk (ISAR). The multiple estimation of risk based on the Emergency Department Spanish Score in Patients with Acute Heart Failure (MEESSI-AHF risk score) was recorded. Primary Outcome: in-hospital and within 30-day post-discharge mortality.

**Results:**

457 patients were included (mean age 88.60 ± 4.40 years; women 64.3%). Baseline frailty prevalence was high across all instruments (FI-VIG 84.7%, TFI 64.1%, ISAR 62.93%, CFS 60.8%, FRAIL 40.9%). In-hospital and 30-days post-discharge mortality were 8.8% and 13.0% respectively. All frailty instruments assessed at baseline and admission were associated with in-hospital mortality in univariate analyses. In multivariable models, admission frailty assessed by CFS (OR 2.26, 95% CI 1.21–4.21, *p* = 0.010) and TFI (OR 1.68, 95% CI 1.23–2.28 *p* < 0.001), together with the MEESSI-AHF score 1.42, 95% CI 1.02–1.97 *p* = 0.036), remained independently associated with in-hospital mortality. Frailty assessed at discharge emerged as the strongest predictor of 30-day post-discharge mortality, with FI–VIG (OR 1.75, 95% CI 1.35–2.29 *p* < 0.001), TFI (OR 1.24, 95% CI 1.01–1.52 *p* = 0.040), and MEESSI-AHF score (OR 1.22, 95% CI 1.05–1.41 *p* = 0.008) remaining independently associated.

**Conclusions:**

Frailty is highly prevalent and dynamically worsens during AHF hospitalization in older patients. Admission frailty identifies patients at risk of in-hospital death, whereas frailty at discharge captures residual vulnerability associated with early post-discharge mortality. Incorporating dynamic frailty assessment may improve prognostic stratification in AHF.

## Background

Frailty is defined as an increased vulnerability to endogenous and exogenous stressors resulting from a decline in homeostatic reserves [1]. It represents a dynamic state from the complex interaction of physical, cognitive, psychological, nutritional and social factors, which compromises the ability of the older adults to respond to acute stressors. Frailty has been consistently associated with disability, adverse events, prolonged hospital stay, readmissions, falls, and mortality, and particularly among hospitalized older patients experiencing acute illness [2].

The prevalence of frailty varies widely depending on the assessment instrument used and the healthcare setting. In community-dwelling older adults in Spain, frailty affects 15–26% of individuals aged 80–84 years and 18–38% of those aged *≥* 85 years [3]. In contrast, among older adults admitted to acute care hospitals, frailty prevalence ranges from 34% to 45%, according to systematic reviews and meta-analyses [4, 5]. Importantly, frailty has been described as a dynamic condition that may worsen during hospitalization, with moderate-to-severe frailty affecting up to 79.6% of patients in acute care settings and being more prevalent in medical than surgical wards [5–7].

There is a wide range of tools to measure the frailty, which are broadly based on two conceptual models: the unidimensional physical phenotype and the multidimensional cumulative deficit approach that integrates the complex interactions of the physical, psychological, and social domains [8]. Some instruments, such as the Frailty Index (FI), Clinical Frailty Scale (CFS), FRAIL scale, and Tilburg Frailty Indicator (TFI), are commonly used in clinical practice as screening tools to identify vulnerable individuals, whereas the physical frailty phenotype is more often considered a diagnostic framework [2, 8]. In addition, frailty is increasingly recognized as a multidimensional construct extending beyond physical impairment alone. In this context, multidimensional tools may provide a more comprehensive assessment of vulnerability in older adults. However, although physical phenotype models remain influential, their applicability in hospitalized older adults is limited, as acute illness, delirium, malnutrition, secondary sarcopenia and functional decline may influence performance-based measurements such as gait speed or grip strength and do not accurately reflect the real frailty state [2, 9].

Despite the recognized prognostic value of frailty, there is increasing recognition of the importance of frailty assessment in acute care. Recent European expert recommendations [10] support the use of practical tools such as the CFS in emergency settings. However, variability in the choice of instruments across different clinical contexts still exists [2].

Frailty frequently coexists with heart failure (HF), with prevalence estimates ranging from approximately 20% in ambulatory older adults to over 80% in hospitalized patients, depending on the clinical setting and assessment method. Across this spectrum, frailty is consistently associated with increased mortality, hospital admissions, and adverse outcomes in both chronic and acute decompensated HF [11–14]. HF remains the leading cause of hospitalization among very old adults, accounting for nearly half of admissions in octogenarians and over 60% in nonagenarians [15].

Recent evidence suggest that multidimensional frailty assessments may outperform physical frailty tools in predicting mortality among older patients with HF [16–19]. However, single-time-point assessments may fail to capture the dynamic nature of frailty during acute hospitalization. Although prognostic tools such as the multiple estimation of risk based on the Emergency Department Spanish Score in Patients with Acute Heart Failure (MEESSI-AHF risk score) was incorporated to provide robust estimates of short-term mortality [20], frailty remains insufficiently integrated into HF risk stratification models.

To date, prospective studies evaluating transitions in frailty status during hospitalization and their prognostic implications in older patients admitted for acute heart failure (AHF) are scarce.

### Objective of the study

The aim of this study was to compare the performance of different multidimensional frailty instruments (FI-VIG, TFI, CFS and FRAIL) in identifying frailty among older adults hospitalized for AHF, and to evaluate their ability–when assessed at different time points–to predict in-hospital and 30-day post discharge mortality.

## Methods

### Design and participants

The study has a prospective observational cohort of patients (Hospitalized Older Patients with Acute Heart Failure (HOP-AHF Cohort) admitted in the Geriatric Acute Care, Internal Medicine and Cardiology Wards of the University Hospital of Vic, Catalonia (Spain). Data were collected from January 2023 to February 2024, with one month follow-up (March 2024).

Inclusion criteria: patients aged 80 or older admitted in the Geriatric Acute Care, Internal Medicine and Cardiology Wards with a diagnosis of AHF were eligible according to the European Society of Cardiology (symptoms, signs, NT-proBNP > 125 ng/ml, transthoracic echocardiogram findings) [21]. Patients who did not meet these criteria or who did not want to participate in the study were excluded.

Ethics approval: The study was approved by the Ethics Committee of the University Hospital of Vic, FORES (Fundació d’Osona per la Recerca i l’Educació Sanitàries) under the reference number 2,023,628/EO169. We obtained written informed consent from the patient, or in cases of incapacity, dementia or delirium, from the main caregiver.

The primary outcomes were in-hospital and 30-day post-discharge mortality to determinate the best timing to measure frailty as a predictor of mortality and other adverse health outcomes.

### Data collection

All assessments were performed by trained interviewers, including of one geriatric and one internal medicine senior residents. Both assessors had prior clinical experience in the care of older patients and received specific training in the use of frailty instruments before study initiation to ensure standardized data collection. Recruitment process and initial data collection occurred within the first 24–48 h after admission. Functional and frailty measures were obtained at three time points: baseline (defined as the patient´s condition one month prior to hospital admission, based on patient or caregiver report and clinical records when available), within the first 48 h hospital after admission and on the day of discharge. Follow-up was conducted through review of the electronic medical records up to 30 days after discharge.

The study variables in our study were:

- *Sociodemographic data*: age and sex.
- *Social situation*: living at home (alone or with relatives), living in a nursing home.
- *Functionality*: was assessed using the Barthel index (BI) [22] and NYHA functional Classification [23]. The BI measures performance in basic activities of daily living and was categorized as follows: severe dependence (score < 40), assisted independence or moderate dependence (score 40–60), and independence/mild dependence (score > 60). NYHA classification was used to grade functional limitation in patients with HF (I–IV), according to standard criteria published by the New York Heart Association.
- *Comorbidities*: hypertension, type 2 diabetes mellitus, dyslipidaemia, ischemic cardiopathy, atrial fibrillation, peripheral artery diseases, chronic lung disease, chronic renal disease, cancer, orthopaedic disorder, prior stroke, dementia. Comorbidities were selected based on their clinical relevance in older patients with HF, their known association with adverse outcomes, and their availability in routine clinical records.
- *Polypharmacy*: regular use of *≥* 5 medications per day.
- *Heart failure characteristics*: (i) aetiology based on transthoracic echocardiogram and electrocardiogram findings (ii) number of AHF decompensations in the last year (iii) classification according to grade left ventricular ejection fraction (LVEF).
- *Analytical data at admission*: (i) N-terminal pro–B-type natriuretic peptide (NT-proBNP, ng/mL) (ii) Glomerular filtration ratio (GFR, mL/min).
- *Frailty measurements*: Frailty was assessed using four previously published and validated instruments at baseline, within 48 h of admission, and at discharge. We used the following tools: (i) The *Frail-VIG Index* [24], a multidimensional score used in Catalonia, including 22 items assessing 25 deficits. The index is calculated as the number of accumulated deficits divided by the total of potential deficits (range 0.0–1.0). The degree of frailty is categorized as: non-frailty (< 0.20), mild (0.2–0.35), moderate (0.35–0.50) and severe frailty (> 0.5) [25]. (ii) The *Clinical Frailty Scale* (CFS) [26] is based on clinical evaluation of physical activity and functional status. It categorizes the patient using descriptors and pictograms to assess the level of vulnerability, with a range between 1 (very fit), 5 (mild) to 8 (very severe) and 9 (terminally ill). Each increment in one category significantly increases the medium-term risks of mortality and institutionalization; (iii) The *FRAIL* scale [27] comprises components of fatigue, resistance, ambulation, illness, and loss of weight, as captured on a questionnaire (range 0–5); a score *≥* 3 is considered indicative of frailty; (iv) *TFI* [28] comprises two parts: one addressing sociodemographic characteristics and the other addressing components of frailty across physical, psychological, and social domains (score range 0–15), with a score *≥* 5 defining frailty. These instruments were applied in the context of acute hospitalization, acknowledging that some components may be influenced by the patient´s acute clinical condition. Each instrument was applied independently according to its original scoring methodology. These instruments were not originally designed to capture short-term changes over brief periods; therefore, observed variations should be interpreted with caution.
- *Prognostic scales*: (i) ISAR score [29], a six-item self-report screening questionnaire with dichotomous responses (yes/no). A score *≥* 2 points indicate increased risk of future adverse outcomes including frailty, functional decline, unplanned hospitalisation or emergency department (ED) revisits, institutionalization, or death [30]; ii) MEESSI-AHF risk score [20], which stratifies older patients with AHF presenting to the ED according to their 30-day mortality risk. This score is calculated as a weighted sum of 13 items, with weights derived from regression coefficients obtained from multiple regression models. Patients are categorized into four risk groups–low, intermediate, high and very high-risk–reflecting an increasing risk of short-term adverse outcomes related to HF.
- *Discharge destination*: home, nursing home, medium-term care facility.

### Statistical analyses

Continuous variables are presented as means and standard deviations (SD) for normally distributed data, or as medians with 25th and 75th percentiles for non-normally distributed variables. Categorical variables are expressed as frequencies and percentages based on available data.

Associations between clinical and demographic characteristics and mortality were assessed using the chi-square test for categorical variables, the Student’s *t* test for normally distributed continuous variables, and the Mann–Whitney *U* test for non-normally distributed continuous variables. Differences in frailty scale scores according to mortality status were also evaluated using the Student’s *t* test.

Finally, the association between in-hospital and 30-day mortality risk and the different frailty scales was examined using univariable and multivariable logistic regression models. Variables included in the multivariable models were selected based on their clinical relevance and results from univariate analyses.

All statistical analyses were performed using IBM SPSS Statistics version 31. A two-sided *p* value < 0.05 was considered statistically significant.

## Results

### Baseline characteristics

**Fig. 1:**
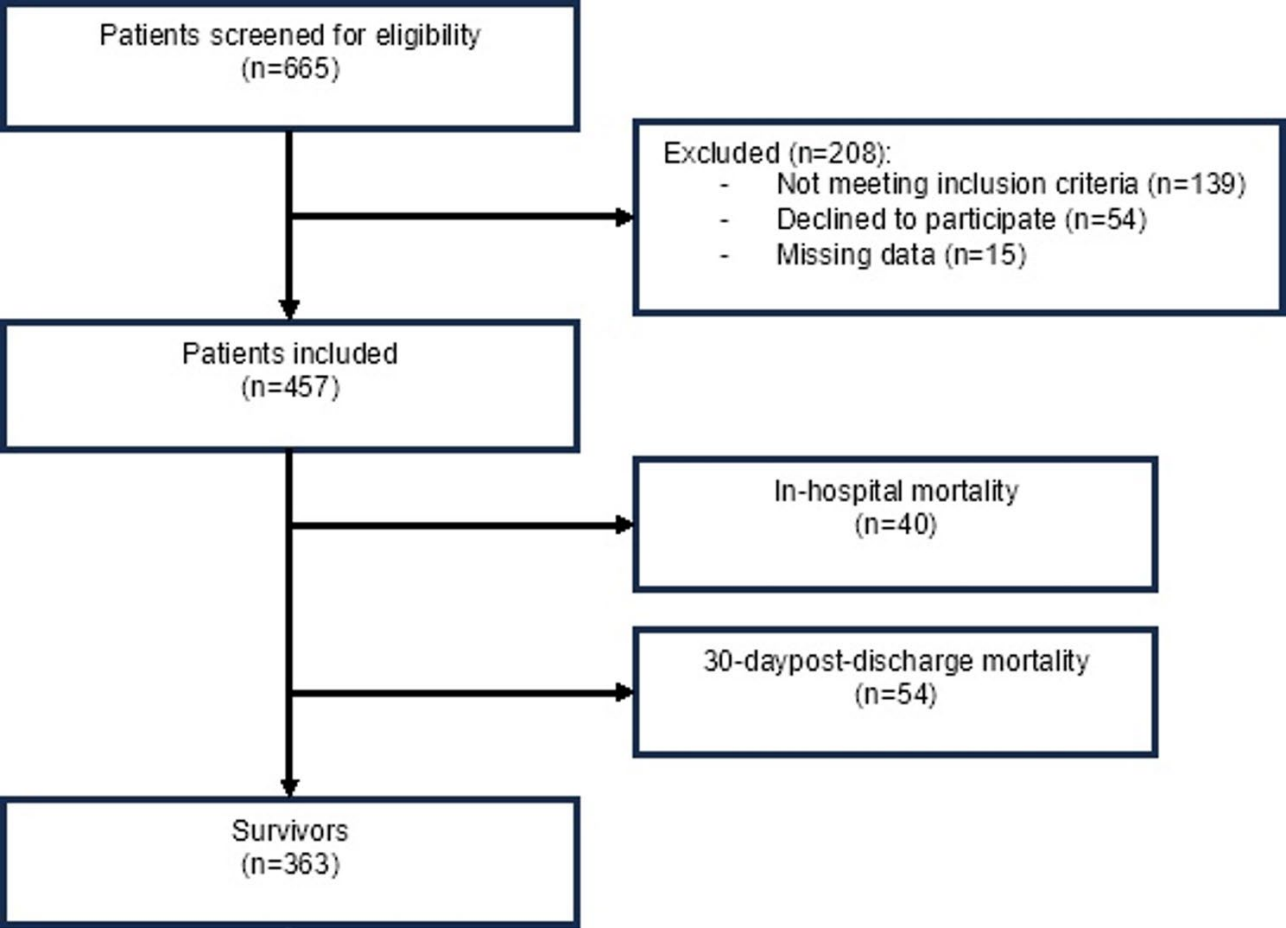
Recruitment process flowchart

A total of 665 patients were screened, of whom 457 patients were included in the final analysis. The reasons for exclusion are detailed in Fig. 1. The mean age was 88.6 ± 4.4 years. Females accounted for 64.3% (*n* = 294) and males 35.7% (*n* = 163). Table 1 summarizes the main demographic and clinical characteristics of the study population according to survival status and total mortality. Of all participants, 78.1% (*n* = 356) lived at home, 19.7% (*n* = 90) in a nursing home, and 2.2% (*n* = 10) in medium-term care prior to admission. The mean length of AHF stay in the overall sample was 7.7 ± 5.8 days, with a mean of 1.25 ± 1.40 AHF decompensations in the last year.

**Table 1:** Baseline characteristic of the study population according to survival status

Demographics	All participants (*n* = 457)	Survivors (*n* = 363, 79.43%)	Total Mortality (*n* = 94, 20.56%)	*P* value
Age (years), mean ± SD	88.6 ± 4.4	88.5 ± 4.3	89.1 ± 4.6	0.238
Sex				
Women, n (%)	294 (64.3)	236 (65.0)	58 (61.7)	0.550
Men, n (%)	163 (35.7)	127 (35.0)	36 (38.3)	
Living situation				
Home	356 (78.1)	291 (80.4)	65 (69.1)	0.064
Nursing Home	10 (2.2)	7 (1.9)	3 (3.2)	
Medium-term Care	90 (19.7)	64 (17.7)	26 (27.7)	
Length of stay, days, median (IQR)	7 (5–9.8)	7 (5–10)	7 (4–9.3)	0.984
Number of medications per day, mean ± SD	9.89 ± 4.2	9.90 ± 3.8	9.86 ± 4.2	0.938
*Comorbidity*				
Hypertension	398 (87.1)	319 (87.9)	79 (84.0)	0.323
Orthopaedic disorder	287 (62.8)	226 (62.3)	61(64.9)	0.638
Atrial Fibrillation	249 (54.7)	195 (53.9)	54 (58.1)	0.468
Dyslipidaemia	196 (42.9)	156 (43.0)	40 (42.6)	0.941
Chronic Renal disease	183 (40.0)	144 (39.7)	39 (41.5)	0.748
Chronic Lung disease	143 (31.3)	119 (32.8)	24 (25.5)	0.177
Diabetes mellitus	141 (30.9)	112 (30.9)	29 (30.9)	1.000
Dementia	132 (28.9)	98 (27.0)	34 (36.2)	0.080
Ischemic cardiopathy	106 (23.2)	83 (22.9)	23 (24.5)	0.743
Cancer	75 (16.4)	57 (15.7)	18 (19,1)	0.421
Prior stroke	67 (14.7)	50 (13.8)	17 (18.1)	0.292
Peripheral Artery diseases	49 (10.7)	38 (10.5)	11 (11.7)	0.730
*HF Etiology*				
Multifactorial	302 (66.1)	244 (67.2)	58 (61.7)	0.314
Valvular heart disease	269 (59.1)	220 (60.9)	49 (52.1)	0.122
Atrial fibrillation	244 (53.4)	191 (52.6)	53 (56.4)	0.514
Hypertensive	226 (49.5)	180 (49.6)	46 (48.9)	0.910
Ischaemic	112 (24.5)	86 (23.7)	26 (27.7)	0.425
Pulmonary arterial hypertension	102 (22.3)	89 (24.5)	13 (13.8)	**0.027**
Unknown	20 (4.4)	19 (5.2)	1 (1.1)	0.092
*Echocardiography*				
LVEF > 50%	343 (75.4)	278 (76.8)	65 (69.9)	0.077
LVEF 41–49%	67 (14.7)	53 (14.6)	14 (15.1)	
LVEF < 40%	45 (9.9)	31 (8.6)	14 (15.1)	
Last year AHF decompensations, median (IQR)	1 (0–2)	1 (0–2)	1 (0–3)	**< 0.001**
Troponin T, pg/mL, median (IQR)	39 (25–65)	37.3 (23.1–60.09)	54.0 (34.0–100.5)	**< 0.001**
NT-proBNP, ng/mL, median (IQR)	4972 (2297.5–11781)	4416 (1991–10300)	10,942 (4063–19806)	**< 0.001**
GFR, mL/min, mean ± SD	46.4 ± 21.3	47.8 ± 21.1	41.2 ± 21.5	**0.007**
*Functional scales*				
NYHA_0_	1.51 ± 0.6	1.47 ± 0.6	1.69 ± 0.6	**< 0.001**
NYHA_1_	3.33 ± 0.6	3.23 ± 0.6	3.69 ± 0.5	**< 0.001**
BI_0_	72.7 ± 21.8	73.92 ± 21.11	62.75 ± 25.08	**< 0.001**
BI_1_	32.8 ± 18.8	34.17 ± 18.05	18 ± 10.84	**< 0.001**
*Frailty Scales*				
FI-VIG_0_	0.32 ± 0.13	0.31 ± 0.12	0.37 ± 0.12	**< 0.001**
FI-VIG_1_	0.51 ± 0.13	0.48 ± 0.12	0.62 ± 0.11	**< 0.001**
FRAIL_0_	2.11 ± 1.1	1.98 ± 1.1	2.59 ± 1.1	**< 0.001**
FRAIL_1_	3.75 ± 0.8	3.67 ± 0.7	4.06 ± 0.7	**< 0.001**
CFS_0_	4.83 ± 1.3	4.70 ± 1.3	5.35 ± 1.3	**< 0.001**
CFS_1_	7.29 ± 1.2	7.07 ± 1.1	8.15 ± 1.0	**< 0.001**
TFI_0_	5.39 ± 2.3	5.15 ± 2.2	6.34 ± 2.2	**< 0.001**
TFI_1_	8.68 ± 2.2	8.25 ± 2.0	10.41 ± 1.9	**< 0.001**
*Prognostic scales*				
MEESSI-AHF score, %, median (IQR)	13.03 (7.08–30.45)	10.95 (6.47–20.29)	36.26 (19.86–55.10)	**< 0.001**
ISAR	2.99 ± 1.2	2.85 ± 1.2	3.55 ± 1.4	**< 0.001**
*Discharge destination*				
Home	188 (45.1)	174 (47.9)	14 (25.9)	**< 0.001**
Medium-term care	166 (39.8)	128 (35.3)	38 (70.4)	
Home hospitalization	63 (15.1)	61 (16.8)	2 (3.7)	

Bold values indicate statistically significant results (*p* < 0.05)

*HF* Heart Failure, *LVEF* Left ventricular ejection fraction, *NT-BNP* Brain natriuretic peptide, *GFR* Glomerular filtration rate, *NYHA*_*0*_ New York Heart Association (one month before admission), *NYHA*_*1*_ New York Heart Association (within the first 48 hours after admission), *BI*_*0*_ Barthel Index (one month before admission), *BI*_*1*_ Barthel Index (within the first 48 hours after admission), *FI-VIG*_*0*_ Frail Index-VIG (one month before admission), *FI-VIG*_*1*_ Frail Index-VIG (within the first 48 hours after admission), *FRAIL*_*0*_ FRAIL scale (one month before admission), *FRAIL*_*1*_ FRAIL scale (within the first 48 hours after admission), *CFS*_*0*_ Clinical Frailty Scale (one month before admission), *CFS*_*1*_ Clinical Frailty Scale (within the first 48 hours after admission), *TFI*_*0*_ Tilburg Frailty Indicator (one month before admission), *TFI*_*1*_ Tilburg Frailty Indicator (within the first 48 hours after admission), *MEESSI-AHF score* Multiple Estimation of Risk based on the Emergency Department Spanish Score in Patients with Acute Heart Failure, *ISAR* Identification of Seniors at Risk

*Values are presented as mean standard deviation or median (interquartile range), as appropriate. Comparisons were performed using Student´s T-test, Mann-Whitney U test, or chi-square test

Among the cohort, the most frequent comorbidities were hypertension (87.1%, *n* = 398), orthopaedic disorder (62.8%, *n* = 287), atrial fibrillation (54.7%, *n* = 249), dyslipidaemia (42.9%, *n* = 196), and chronic renal disease (40%, *n* = 183). Polypharmacy was detected in 91.24% of patients (*n* = 417), with a mean 9.89 ± 4.2 medications per day.

The main causes of HF were multifactorial aetiology (66.1%, *n* = 302), following by valvular heart diseases (59.1%, *n* = 269), and atrial fibrillation (53.4%, *n* = 244). Most patients (75.4%, *n* = 343) had preserved left ventricular ejection fraction on transthoracic echography. Laboratory findings included a mean glomerular filtration rate of 46.40 ± 21.33 ml/min, NT-proBNP of 4972 ng/mL (IQR 2297.5–11781), and troponin of 39 pg/mL (IQR 25–65).

Following discharge, 45.1% (*n* = 188) returned home, 39.8% (*n* = 166) required medium-term care, and 15.1% (*n* = 63) received home-based hospitalization. Almost one third of the sample (35.7%, *n* = 148) were readmitted within 30 days of discharge.

### Functionality transitions

Participants showed mild pre-admission dependence in basic activities in daily live (72.70 ± 21.80 as BI baseline). Following admission of AHF, functional domains markedly declined to 32.80 ± 18.80, and partially recovered at discharge, with a mean of BI 53.34 ± 23.76 among survivors. Similarly, NYHA increased from 1.51 ± 0.60 at baseline to 3.33 ± 0.60 at admission, improving to 2.21 ± 0.71 at discharge.

### Frailty trajectory in survivors

At baseline, frailty prevalence was high across all instruments: FI-VIG 84.68% (*n* = 387), TFI 64.11% (*n* = 293), CFS 60.83% (*n* = 278), and FRAIL 40.91% (*n* = 187). The mean baseline frailty severity was 0.32 ± 0.13 (FI-VIG_0_), 5.39 ± 2.3 (TFI_0_), 4.83 ± 1.3(CFS_0_), and 2.11 ± 1.11 (FRAIL_0_). Within the first 48 h after admission, frailty worsened substantially, with mean values of 0.51 ± 0.13 (FI-VIG_1_), 8.68 ± 2.20 (TFI_1_), 7.29 ± 1.2 (CFS_1_), and 3.75 ± 0.80 (FRAIL_1_).

Table 2 summarizes the frailty trajectories experienced by survivors during hospitalization and 30 days follow-up after discharge, comparing frail and non-frail patients. For the FI-VIG, 71.62% of patients (*n* = 260; 0.36 ± 0.09) were mildly or moderately frail at baseline, whereas 28.37% (*n* = 103; 0.16 ± 0.04) were non-frail. Within the first 48 h of admission, the proportion of patients classified as moderate–severe frailty increased to 98.34% (*n* = 357; 0.49 ± 0.12). At discharge, most patients remained in the moderate–severe frailty category (92.28%; *n* = 335; 0.43 ± 0.12; *p* < 0.001), while only 7.71% were non-frail (*n* = 28; 0.17 ± 0.03).

**Table 2:** Frailty trajectory among hospital survivors according to frail and non-frail status

Time point	FI-VIG			FRAIL			CFS			TFI			
	All	Frail	Non-frail	All	Frail	Non-frail	All	Frail	Non-frail	All	Frail	Non-frail	
Baseline	0.31 ± 0.12 (*n* = 363)	0.36 ± 0.09 (*n* = 260)	0.16 ± 0.04 (*n* = 103)	1.98 ± 1.09 (*n* = 363)	4.00 ± 0.00 (*n* = 20)	1.87 ± 1.03 (*n* = 343)	4.70 ± 1.34 (*n* = 363)	6.33 ± 0.51 (*n* = 105)	4.03 ± 0.94 (*n* = 258)	5.15 ± 2.21 (*n* = 363)	7.30 ± 1.32 (*n* = 149)	3.66 ± 1.27 (*n* = 214)	
Admission	0.48 ± 0.12 (*n* = 363)	0.49 ± 0.12 (*n* = 357)	0.19 ± 0.02 (*n* = 6)	3.67 ± 0.74 (*n* = 363)	4.27 ± 0.45 (*n* = 197)	2.96 ± 0.19 (*n* = 166)	7.07 ± 1.09 (*n* = 363)	7.23 ± 0.85 (*n* = 343)	4.30 ± 1.13 (*n* = 20)	8.25 ± 1.99 (*n* = 363)	8.58 ± 1.71 (*n* = 332)	4.68 ± 0.98 (*n* = 31)	
Discharge	0.41 ± 0.14 (*n* = 363)	0.43 ± 0.12 (*n* = 335)	0.17 ± 0.03 (*n* = 28)	2.96 ± 1.04 (*n* = 363)	4.20 ± 0.40 (*n* = 104)	2.46 ± 0.77 (*n* = 259)	5.85 ± 1.39 (*n* = 363)	6.68 ± 0.79 (*n* = 234)	4.35 ± 0.87 (*n* = 129)	6.76 ± 2.23 (*n* = 363)	7.84 ± 1.63 (*n* = 257)	4.14 ± 0.91 (*n* = 106)	

*Values are expressed as mean ± standard deviation. Frailty classification was based on scale-specific validated cut-off points

*Abbreviations*: *FI-VIG* Frail-VIG Index, *FRAIL* FRAIL scale, *CFS* Clinical Frailty Scale, *TFI* Tilburg Frailty Indicator

For the FRAIL scale, 5.50% (*n* = 20; 4.00 ± 0.00) were frail at baseline, while 94.49% (*n* = 343; 1.87 ± 1.03) were non-frail/prefrail. At admission, 54.26% (*n* = 197; 4.27 ± 0.45) were frail. By discharge, 28.65% (*n* = 104; 4.20 ± 0.40) remained frail, whereas 71.34% (*n* = 259; 2.46 ± 0.77) were non-frail.

For the CFS, 28.92% (*n* = 105; 6.33 ± 0.51) were frail at baseline, while 71.07% (*n* = 258; 4.03 ± 0.94) were non-frail. Frailty increased substantially during the first 48 h, with 94.49% (*n* = 343; 7.23 ± 0.85) meeting frailty criteria. At discharge, 64.46% (*n* = 234; 6.68 ± 0.79) remained frail.

For the TFI, 41.04% of patients (*n* = 149; 7.30 ± 1.32) were frail at baseline, while 58.95% (*n* = 214; 3.66 ± 1.27) were non-frail. At 48 h, frailty prevalence increased to 91.46% (*n* = 332; 8.58 ± 1.71). At discharge, 70.79% (*n* = 257; 7.84 ± 1.63) remained frail.

The median MEESSI-AHF risk score of survivors was 10.95% (IQR 6.47–20.79), corresponding to the high-risk category.

### Prognostic scales and mortality trajectories between frailty tools

Overall, 8.8% (*n* = 40) patients died during the hospitalization and 13% (*n* = 54) 30 days after medical discharge. The mean ISAR score was 2.99 ± 1.23. Among survivors, the mean score was 2.85 ± 1.20, whereas patients who died had significantly higher scores (3.55 ± 1.40; *p* < 0.001), indicating a higher risk of short-term adverse outcomes in older adults. Although we attempted to perform dynamic ISAR assessments at baseline, admission, and discharge, these repeated measurements did not provide additional prognostic value. The median MEESSI-AHF risk score for the overall cohort was 13.03% (IQR 7.08–30.45), corresponding to an intermediate-risk category. In contrast, patients who died had a median score of 36.26% (IQR 19.86–55.10), indicating a very high risk of AHF mortality.

The frailty scores differed between participants who died in-hospital and those who died within 30 days after medical discharge (Table 3). At baseline, frailty severity was comparable between groups across all instruments, with no statistically significant differences. At admission, frailty scores increased markedly with significantly higher scores among patients who died in-hospital compared with those who died after discharge, particularly for FI-VIG (0.65 ± 0.10 vs. 0.59 ± 0.11, *p* = 0.009), CFS (8.55 ± 0.68 vs. 7.85 ± 1.04, *p* < 0.001), and TFI (10.89 ± 1.98 vs. 10.07 ± 1.78, *p* = 0.041). No significant differences were observed using FRAIL scale at any time point. At discharge, frailty remained markedly elevated at discharge in the 30-day mortality group across all instruments. These results indicate the higher frailty at admission was associated with in-hospital mortality, whereas persistent frailty at discharge characterized patients at increased risk of early post-discharge mortality.

**Table 3:** Comparison of frailty scale scores according to in-hospital mortality and 30-day post-discharge mortality

Time point	FI-VIG				FRAIL				CFS				TFI			
	All	IHM	30d-M	*p*	All	IHM	30d-M	*p*	All	IHM	30d-M	*p*	All	IHM	30d-M	*p*
Baseline	0.37 ± 0.12 (*n* = 94)	0.36 ± 0.12 (*n* = 40)	0.37 ± 0.12 (*n* = 54)	0.616	2.59 ± 1.08 (*n* = 94)	2.50 ± 1.22 (*n* = 40)	2.65 ± 0.97 (*n* = 54)	0.514	5.35 ± 1.25 (*n* = 94)	5.48 ± 1.36 (*n* = 40)	5.26 ± 1.17 (*n* = 54)	0.411	6.34 ± 2.20 (*n* = 94)	6.21 ± 2.58 (*n* = 40)	6.43 ± 1.90 (*n* = 54)	0.663
Admission	0.62 ± 0.11 (*n* = 94)	0.65 ± 0.10 (*n* = 40)	0.59 ± 0.11 (*n* = 54)	0.009	4.06 ± 0.72 (*n* = 94)	4.13 ± 0.79 (*n* = 40)	4.02 ± 0.66 (*n* = 54)	0.491	8.15 ± 0.96 (*n* = 94)	8.55 ± 0.68 (*n* = 40)	7.85 ± 1.04 (*n* = 54)	< 0.001	10.41 ± 1.90 (*n* = 94)	10.89 ± 1.98 (*n* = 40)	10.07 ± 1.78 (*n* = 54)	0.041
Discharge	0.58 ± 0.12 (*n* = 54)	-	0.58 ± 0.12 (*n* = 54)	-	3.63 ± 0.85 (*n* = 54)	-	3.63 ± 0.85 (*n* = 54)	-	7.33 ± 1.33 (*n* = 54)	-	7.33 ± 1.33 (*n* = 54)	-	9.04 ± 1.89 (*n* = 54)	-	9.04 ± 1.89 (*n* = 54)	-

*In-hospital mortality group is not applicable at discharge, as only survivors were assessed at this time point

*Abbreviations*:*IHM* In-hospital mortality, *30d-M* 30-day post-discharge mortality, *FI-VIG* Frailty-VIG Index, *FRAIL* FRAIL scale, *CFS* Clinical Frailty Scale, *TFI* Tilburg Frailty Indicator

All four frailty instruments—at both baseline and admission—were significantly associated with in-hospital mortality (Table 4). The admission frailty showed the strongest associations, particularly CFS1 (OR 6.34, 95% CI 3.71–10.86; *p* < 0.001), FRAIL1 (OR 2.06, 95% CI 1.33–3.21; *p* < 0.001), FI-VIG1 (OR 1.63, 95% CI 1.40–1.90; *p* < 0.001), and TFI1 (OR 1.86, 95% CI 1.52–2.27; *p* < 0.001). The MEESSI-AHF score also remained independently associated with in-hospital mortality (OR 2.11, 95% CI 1.46–3.05; *p* < 0.001).

**Table 4:** Association of frailty measures and MEESSI-AHF score with in-hospital mortality

	Univariate OR (95% CI)	*p*-value	Multivariate OR (95% CI)	*p*-value
FI-VIG_0_	1.12 (1.01–1.25)	0.032	-	-
FI-VIG_1_	1.63 (1.40–1.90)	< 0.001	1.20 (0.98–1.46)	0.076
FRAIL_0_	1.44 (1.06–1.97)	0.020	-	-
FRAIL_1_	2.06 (1.33–3.21)	< 0.001	-	-
CFS_0_	1.52 (1.17–1.99)	0.002	-	-
CFS_1_	**6.34 (3.71–10.86)**	**< 0.001**	**2.26 (1.21–4.21)**	**0.010**
TFI_0_	1.19 (1.03–1.38)	0.021	0.73 (0.57–0.92)	**0.007**
TFI_1_	**1.86 (1.52–2.27)**	**< 0.001**	**1.68 (1.23–2.28)**	**< 0.001**
MEESSI-AHF score	**2.11 (1.46–3.05)**	**< 0.001**	**1.42 (1.02–1.97)**	**0.036**

Odds ratios (ORs) with 95% confidence intervals (CIs) are shown. Variables included in the multivariable models were selected based on clinical relevance and univariate analyses

Bold values indicate statistically significant results (*p* < 0.05)

*Abbreviations*: *FI-VIG*_*0*_ Frail Index-VIG assessed one month before admission, *FI-VIG*_*1*_ Frail Index-VIG assessed within the first 48 hours after admission, *FRAIL*_*0*_ FRAIL scale assessed one month before admission, *FRAIL*_*1*_ FRAIL scale assessed within the first 48 hours after admission, *CFS*_*0*_ Clinical Frailty Scale assessed one month before admission, *CFS*_*1*_ Clinical Frailty Scale assessed within the first 48 hours after admission, *TFI*_*0*_ Tilburg Frailty Indicator assessed one month before admission, *TFI*_*1*_ Tilburg Frailty Indicator assessed within the first 48 hours after admission, *MEESSI-AHF score* Multiple Estimation of Risk based on the Emergency Department Spanish Score in Patients with Acute Heart Failure

In multivariable analysis, admission frailty assessed by CFS1 (OR 2.26, 95% CI 1.21–4.21; *p* = 0.010) and TFI1 (OR 1.68, 95% CI 1.23–2.28; *p* < 0.001) remained independently associated with in-hospital mortality, whereas FI-VIG1 showed a borderline association. Baseline TFI was inversely associated with mortality (OR 0.73, 95% CI 0.57–0.92; *p* = 0.007), and the MEESSI-AHF score retained independent prognostic value (OR 1.42, 95% CI 1.02–1.97; *p* = 0.036).

Regarding 30-day post-discharge mortality (Table 5), frailty assessed at discharge emerged as the strongest independent predictor. In multivariant models, FI-VIG2 (OR 1.75, 95% CI 1.35–2.29; *p* < 0.001), TFI2 (OR 1.24, 95% CI 1.01–1.52; *p* = 0.040) and MEESSI-AHF score (OR 1.22, 95% CI 1.05–1.41); *p* = 0.008) remained independently associated with mortality, whereas baseline and admission measures did not retain significance.

**Table 5:** Association of frailty measures and the MEESSI-AHF score with 30-day post-discharge mortality

	Univariate OR (95% CI)	*p*-value	Multivariate OR (95% CI)	*p*-value
FI-VIG_0_	1.19 (1.08–1.31)	< 0.001	0.83 (0.71–0.97)	0.018
FI-VIG_1_	1.38 (1.24–1.55)	< 0.001	0.77 (0.60–0.98)	0.035
FI-VIG_2_	1.52 (1.35–1.71)	< 0.001	1.75 (1.35–2.29)	< 0.001
FRAIL_0_	1.87 (1.38–2.53)	< 0.001	-	-
FRAIL_1_	1.89 (1.27–2.78)	0.002	-	-
FRAIL_2_	2.05 (1.48–2.85)	< 0.001	-	-
CFS_0_	1.40 (1.11–1.75)	0.004	-	-
CFS_1_	2.31 (1.65–3.23)	< 0.001	-	-
CFS_2_	2.44 (1.87–3.20)	< 0.001	-	-
TFI_0_	1.31 (1.14–1.49)	< 0.001	-	-
TFI_1_	1.66 (1.39–1.96)	< 0.001	-	-
TFI_2_	1.62 (1.39–1.89)	< 0.001	1.24 (1.01–1.52)	0.040
MEESSI-AHF score	1.44 (1.28–1.62)	< 0.001	1.22 (1.05–1.41)	0.008

*Abbreviations*: *FI-VIG*_*0*_ Frail Index-VIG assessed one month before admission, *FI-VIG*_*1*_ Frail Index-VIG assessed within the first 48 h after admission, *FI-VIG*_*2*_ Frail Index-VIG assessed at medical discharge, *FRAIL*_*0–2*_ FRAIL scale assessed at the corresponding time points, *CFS*_*0– 2*_ Clinical Frailty Scale assessed at the corresponding time points, *TFI*_*0–2*_ Tilburg Frailty Indicator assessed at the corresponding points, *MEESSI-AHF score* Multiple Estimation of Risk based on the Emergency Department Spanish Score in Patients with Acute Heart Failure

Odds ratios (ORs) with 95% confidence intervals (CIs) are shown. Variables included in the multivariable models were selected based on clinical relevance and univariate analyses

These findings highlight the dynamic nature of frailty in AHF, with admission frailty identifying patients at risk of in-hospital death and discharge frailty capturing residual vulnerability associated with early post-discharge mortality.

## Discussion

Our prospective analysis of functional and frailty transitions in older patients hospitalized with AHF highlights the dynamic behaviour of frailty assessments during acute hospitalization. The results show the survival time decreased significantly as frailty worsened during hospitalization with the most frequent transitions occurring at admission and at medical discharge, particularly among patients with moderate frailty at AHF admission.

Flores-Álvarez et al. [12] reported a high prevalence of frailty among older adults with HF and demonstrated its strong association with adverse clinical events and increased healthcare utilization. In the line with these findings, frailty prevalence in our cohort was remarkably high, although it varied substantially depending on the assessment tool used: with the FI-VIG (84.7%), TFI (64.1%), CFS (60.8%), and FRAIL scale (40.9%). This gradient reflects the ability of multidimensional instruments to capture a broader spectrum of vulnerability compared with primarily physical or phenotype-based tools, reinforcing the notion that frailty burden in AHF is highly dependent on the assessment approach. In addition, these differences may also reflect variations in sensitivity, specificity, and item composition across instruments, as the number and type of deficits included can substantially influence frailty prevalence estimates.

Our findings are broadly aligned with those of Kaufmann et al. [31], who demonstrated that frailty at admission is a strong independent predictor of mortality in older patients hospitalized with AHF. In their study, both moderate and severe frailty at admission were associated with increased one-year mortality, and changes in frailty status during hospitalization carried prognostic significance. Similarly, our results emphasize that admission frailty is a relevant marker of short-term outcomes, while dynamic reassessment—particularly at discharge—adds important prognostic mortality prediction beyond baseline status alone.

Importantly, only FI-VIG and CFS predicted in-hospital and short-term mortality in our multivariable models, underscoring that the choice of frailty instrument significantly influences risk stratification. Compared with simpler classification systems [31], the use of multidimensional deficit-accumulation tools in our study likely captured a more comprehensive vulnerability profile, which may explain both the high baseline frailty prevalence and the strong prognostic value of discharge frailty (FI-VIG2) for 30-day post-discharge mortality.

HF severity, as reflected by NYHA class, may also contribute to mortality risk independently of frailty. The relationship between disease severity and frailty is complex, and both factors likely interact to influence clinical outcomes in older patients with AHF.

Patients frequently experienced functional decline and worsening frailty during hospitalization; however, transitions to severe frailty were uncommon over the short inpatient period. Notably, discharge frailty status emerged as the strongest predictor of early post-discharge mortality, supporting the concept that residual vulnerability at discharge reflects an incomplete recovery from the acute event and identifies patients at particularly high risk.

The high rate of 30-day readmissions, together with the incomplete recovery in NYHA class at discharge, suggests that many patients were discharged with residual clinical instability. This may partly explain the elevated risk of early post-discharge mortality observed in our cohort.

Frailty is traditionally conceptualized as a chronic state of increased vulnerability resulting from cumulative decline in physiological reserves. However, in the context of acute illness such as hospitalization for AHF, short-term changes in clinical and functional status may influence frailty assessments, particularly when using instruments that incorporate functional or subjective components. Therefore, some of the variations observed in frailty measures during hospitalization may reflect acute and potentially reversible functional decline rather than true changes in underlying frailty status.

These findings should therefore be interpreted as reflecting dynamic changes in frailty-related measures during acute illness rather than true short-term modifications of the underlying frailty state, although they remain clinically meaningful for risk stratification.

The inverse association observed for baseline TFI in multivariable models deserves consideration. Baseline TFI captures chronic multidimensional vulnerability prior to hospitalization, whereas short-term mortality during acute hospitalization appears to be more strongly driven by acute functional decline and frailty assessed at admission and discharge. This finding suggests that dynamic frailty measures outperform baseline assessments in predicting short-term outcomes in older patients hospitalized with AHF.

Although the ISAR score was associated with mortality in univariate analyses, repeated ISAR assessments did not provide additional prognostic value in dynamic models. This is likely related to its role as a screening tool designed to identify baseline vulnerability rather than to capture short-term changes in frailty during acute illness, further supporting the need for instruments capable of longitudinal assessment in hospitalized older adults.

To our knowledge, no previous studies have integrated multiple frailty instruments alongside the MEESSII-AHF risk score within the same prognostic framework in very old patients hospitalized for AHF. While prior research has demonstrated the prognostic value of these tools individually, evidence regarding their comparative and combined use remains limited. Our findings suggest that incorporating dynamic frailty assessment into existing risk stratification models may enhance prognostic accuracy.

Future studies should compare the predictive performance of different frailty instruments across time points to determine their relative clinical utility in patients with AHF.

This study has several limitations. First, the follow-up period was short, and frailty trajectories beyond 30 days after discharge were not assessed. Second, post-discharge frailty reassessment was not systematically performed, and outcomes were derived from electronic health records. Third, baseline measurements were partially based on patient or caregiver recall, which may introduce recall bias. Fourth, although the FI-VIG has not been specifically validated in HF populations, our objective was not to validate the instrument but to explore the prognostic relevance of multidimensional frailty assessment in this clinical context. Similar approaches have been adopted in previous studies. Fifth, differences in care processes across hospital wards (Geriatrics, Internal Medicine, and Cardiology) were not specifically analyzed and have influenced patient outcomes. Sixth, assessors were not blinded to previous frailty assessments, which may have introduced observer bias. Finally, functional decline, which is highly relevant in older patients, was not included as an outcome in this study and should be considered in future research.

## Conclusions

Early assessment of frailty at hospital admission may help clinicians better stratify risk, allocate healthcare resources, and optimize in-hospital management of older adults with AHF. Our findings indicate that both admission frailty–particularly when assessed using the CFS and FI-VIG–and the MEESSI-AHF score are useful for identifying patients at increased risk of in-hospital mortality.

Importantly, frailty status at discharge, especially when measured using deficit-accumulation models such as the FI-VIG, emerged as the strongest predictor of 30-day post-discharge mortality, highlighting the prognostic relevance of residual vulnerability at the time of discharge.

Serial frailty assessment provides valuable insight into frailty trajectories and the dynamic accumulation of deficits during hospitalization, offering a more accurate estimation of short-term prognosis. This longitudinal approach may support individualized care planning and adjustment of therapeutic goals in hospitalized older patients with AHF.

Future studies in larger, multicentre cohorts are warranted to validate these findings, further explore the clinical utility of frailty trajectories, and determine how best to integrate dynamic frailty assessment into routine AHF care pathways.

## Data Availability

The datasets used and/or analysed during the current study are available from the corresponding author on reasonable request.

## Abbreviations

BI: Barthel Index
CFS: Clinical Frailty Scale
FI: Frailty Index
FI-VIG: Frail Index-VIG
FRAIL: FRAIL scale
GFR: Glomerular filtration rate
ISAR: Identification of Seniors at Risk
LVEF: Left ventricular ejection fraction
MEESSI-AHF score: Multiple Estimation of Risk based on the Emergency Department Spanish Score in Patients with Acute Heart Failure
NT–proBNP: N-terminal pro-brain natriuretic peptide
NYHA: New York Heart Association
TFI: Tilburg Frailty Indicator
